# Proteomic analysis of purified turkey adenovirus 3 virions

**DOI:** 10.1186/s13567-015-0214-z

**Published:** 2015-07-09

**Authors:** Pankaj Kumar, Jan van den Hurk, Lisanework E. Ayalew, Amit Gaba, Suresh K. Tikoo

**Affiliations:** Vaccine and Infectious Disease Organization –International Vaccine Center (VIDO- InterVac1), University of Saskatchewan, Saskatoon, S7N 5E3 SK Canada; Vaccinology & Immunotherapeutics program, School of Public Health, University of Saskatchewan, Saskatoon, S7N 5E5 SK Canada

## Abstract

Turkey adenovirus 3 (TAdV-3) causes high mortality and significant economic losses to the turkey industry. However, little is known about the molecular determinants required for viral replication and pathogenesis**.** Moreover, TAdV-3 does not grow well in cell culture, thus detailed structural studies of the infectious particle is particularly challenging. To develop a better understanding of virus-host interactions, we performed a comprehensive proteomic analysis of proteinase K treated purified TAdV-3 virions isolated from spleens of infected turkeys, by utilizing one-dimensional liquid chromatography mass spectrometry. Our analysis resulted in the identification of 13 viral proteins associated with TAdV-3 virions including a novel uncharacterized TaV3gp04 protein. Further, we detected 18 host proteins in purified virions, many of which are involved in cell-to cell spread, cytoskeleton dynamics and virus replication. Notably, seven of these host proteins have not yet been reported to be present in any other purified virus. In addition, five of these proteins are known antiviral host restriction factors. The availability of reagents allowed us to identify two cellular proteins (collagen alpha-1 (VI) chain and haemoglobin) in the purified TAdV-3 preparations. These results represent the first comprehensive proteomic profile of TAdV-3 and may provide information for illustrating TAdV-3 replication and pathogenesis.

**Electronic supplementary material:**

The online version of this article (doi:10.1186/s13567-015-0214-z) contains supplementary material, which is available to authorized users.

## Introduction

Hemorrhagic enteritis (HE) is an economically important disease of turkeys characterized by depression, splenic enlargement, intestinal haemorrhages and sudden death [1]. The disease is caused by turkey adenovirus 3 (TAdV-3), also known as hemorrhagic enteritis virus (HEV), a member of genus *Siadenovirus A* [2]. Oral infection of susceptible turkeys with pathogenic TAdV-3 strains results in well-characterized splenomegaly and intestinal bleeding in 4 to 6 days causing subclinical infections and mortality [3]. Although TAdV-3 remains one of the most important causes of economic loss to turkey industry, critical molecular determinants of virulence and factors affecting virus replication are not well understood. This may be in part because of unavailability of an efficient “in vitro” tissue culture system for propagation of TAdV-3 [4-6].

The genome of TAdV-3 is 26,263 bp [7]. Although, TAdV-3 genomic organization of central block of genus-common genes [8] appears similar to that of other adenovirus genomes [7], the left (E1) and right (E4) terminal regions appear absent. Interestingly, TAdV-3 encodes a genus specific protein, which shows similarity to bacterial sialidase protein [8]. Although Western blot analysis of purified TAdV-3 particles isolated from crude spleen extract revealed presence of eleven structural polypeptides with apparent molecular weight ranging from 9.5 to 96 kDa [9], no systematic study has been performed to identify the precise protein composition of purified TAdV-3 particles.

In recent years, mass spectrometry (MS) based proteomic characterization has revealed important insights into viral replication, tropism and virulence for a number of different enveloped viruses [10-14]. In contrast, a few proteomic studies have been reported for non-enveloped viruses [15-18]. Additionally, there is now compelling evidence suggesting that host cellular proteins incorporated in the virions play an important role in viral replication and pathogenesis [10,13,19,20]. Using MS based approaches, a number of host proteins have been reported to be incorporated into RNA viruses (“human immunodeficiency virus-1 [10,13]”; “simian immunodeficiency virus [21]”; “respiratory syncytial virus [22]; hepatitis C virus [23]”; “swine hepatitis E virus [24]”; “coronavirus [25]” and “influenza [20,26]”) or DNA viruses (“herpes simplex virus 1 [27]”; “African swine fever virus [28]”; “KSHV [29]”; “Marek’s disease virus (MDV) [30]”, and “mimivirus [31]”). However, to the best of our knowledge, characterization of the host cellular factors integrated into virions for any member of *Adenoviridae* family including TAdV-3 has not been reported so far.

Here, we report the protein composition of the purified TAdV-3 particles by performing a comprehensive proteomic analysis utilizing liquid chromatography-mass spectrometry (LC-MS/ MS). Our analysis resulted in successful identification of 13 viral structural proteins and 18 host-incorporated proteins. Moreover, incorporation of two host proteins in purified virions was verified by Western blot analysis using available immunological reagents.

## Materials and methods

### Turkey and viruses

All turkey procedures were approved by University Committee of Animal Care and Supply (protocol # 19940211) at the University of Saskatchewan, Saskatoon, Canada according to guidelines set by the Canadian Council of Animal Care.

Day-old Hybrid poults obtained from Chinook belt Hatcheries, Calgary, Canada were housed in isolation rooms throughout the experiments. The avirulent TAdV-3 isolate (pheasant origin) was passaged in sero negative turkeys by oral inoculation and purified from crude spleen extracts, as described earlier [32].

### Virion purification

The TAdV-3 virions were purified as previously described [9]. The proteinase K (pK) treatment of purified TAdV-3 virions was performed as described previously [33]. Briefly, double CsCl-purified virions were incubated in 1 mL of MNT buffer (30 mM morpholineethanesulfonic acid [MES], 10 mM NaCl, and 20 mM Tris–HCl [pH 7.4]) containing proteinase K [0 to 20 μg] (Roche, Mannheim, Germany) for 45 min at room temperature and subsequently treated with “2 mM phenylmethyl-sulfonyl fluoride” (Roche) prior to purification by CsCl density gradient centrifugation. Purified virions were resuspended in 10% glycerol and stored at −80 °C until further use. The experiments were performed in triplicate employing three independent virus preparations.

### Negative staining and transmission electron microscopy

Electron microscopy was performed on CsCl_2_ gradient purified TAdV-3 virions (proteinase K treated or untreated) at EM facility at Biology department, University of Victoria, BC, Canada, as described [34]. Briefly, for negatively stained preparation, CsCl_2_ gradient purified virus was first applied onto carbon and formvar coated grids, washed with H_2_0 and stained with 2% aqueous phosphotungstic acid. The specimens were photographed using a charge-coupled device camera (Advanced Microscopy Techniques, AMT CCD camera equipped Hitachi H7000 TEM operating at 75 kv).

### Antibodies

Production and characterization of anti-TAdV-3 serum and monoclonal antibodies (MAbs) recognizing TAdV-3 hexon (15G4) and fiber (87–03) proteins has been described earlier [4,9]. Chicken polyclonal anti-human hemoglobin serum (ab28961) was purchased from Abcam (Cambridge, MA, USA). Rabbit polyclonal anti-human collagen type VI alpha-1 serum (COL6A1) was purchased from antibodies-online Inc. (Atlanta, GA, USA). Alkaline phosphatase conjugated goat anti-rabbit (Sigma Aldrich) and peroxidase-conjugated goat “anti-turkey” IgG (KPL, Maryland, USA) were used as described [4,9].

### Western blotting

Proteins from purified TAdV-3 were separated by sodium dodecyl sulphate (SDS) polyacrylamide gel electrophoresis (PAGE) on 10–15% or 4–15% precast gradient gels (Bio-Rad),transferred to nitrocellulose membrane and probed with protein specific antibodies as described previously [9].

### In solution trypsin digestion

Proteins from CsCl_2_ gradient purified virion-enriched (proteinase K treated or untreated) samples were diluted with 200 mM ammonium bicarbonate prior to reduction with 200 mM dithiothreitol and incubated 30 min at 37 °C. Cysteine sulfhydryl groups were alkylated with 20 μL of 100 mM iodoacetamide (30 min at 37 °C in darkness). Each sample was digested with 5 μg of trypsin (Promega) at 37 °C for 16 h [33,35]. Finally, the samples were de-salted on a Waters HLB Oasis column, speed vac concentrated and stored at −80 °C prior to LC-MS analysis.

#### LC-MS/MS analysis

The peptide mixtures were separated by on-line reverse phase chromatography using a EASY-nLC II system (Thermo Scientific) with a reversed-phase Magic C-18AQ pre-column (100 μm I.D., 2 cm length, 5 μm, 100 Å, Michrom Bio Resources Inc, Auburn, CA, USA) and reversed phase nano-analytical column Magic C-18AQ (75 μm I.D., 15 cm length, 5 μm, 100 Å, Michrom Bio Resources Inc, Auburn, CA, USA) at a flow rate of 300 1/min. The resulting peptides were analyzed by the chromatography system, which was coupled on-line with a LTQ OrbitrapVelos mass spectrometer (Thermo Fisher Scientific, Bremen, Germany) equipped with a NanosprayFlex source (Thermo Fisher Scientific) as described previously [33,35]. The data was acquired with keratin and trypsin peptide mass exclusion lists.

### MS/MS data analysis

Raw files were analysed with Proteome Discoverer 1.4 software suite (Thermo Scientific). Parameters for the spectrum selection to generate peak lists of the collision-induced “dissociation (CID) spectra were activation type: CID”; (s/n cut-off: 1.5; total intensity threshold: 0; minimum peak count: 1; precursor mass: 350–5000 Da). The peak lists were submitted to an in-house Mascot 2.3 server against “the following databases”: Uniprot_Trembl 20111103 (17 651 715 sequences; 5,747,683, 275 residues) and Uniprot-Swissprot 20110104 (523 151 sequences; 184 678 199 residues) all species taxonomy.

Database search parameters were as follows: precursor tolerance 8 ppm; MS/MS tolerance 0.6 Da; Trypsin enzyme 1 missed cleavages; Fourier Transform Ion Cyclotron Resonance (FT-ICR) instrument type; fixed modification: carbamidomethylation (C); variable modifications: deamidation (N,Q); oxidation (M). The Decoy database Percolator settings: Max delta Cn 0.05; Target FDR strict 0.01, Target FDR relaxed 0.05 with validation based on q-Value. Additional virus only species searches were also performed with tolerances previously mentioned. All data were also searched against NCBI (*Gallus gallus* (chicken)) database to detect viral and host proteins. Only sequences identified with a mascot score value greater than 30 were considered as significant. Protein identifications were accepted when the peptide probability was greater than 95.0% [33,35], the protein probability greater than 99.0%, and contained at least 2 identified peptides. Peptide identifications were systemically evaluated manually.

## Results

### Purification of TAdV-3 virions

**Figure 1:**
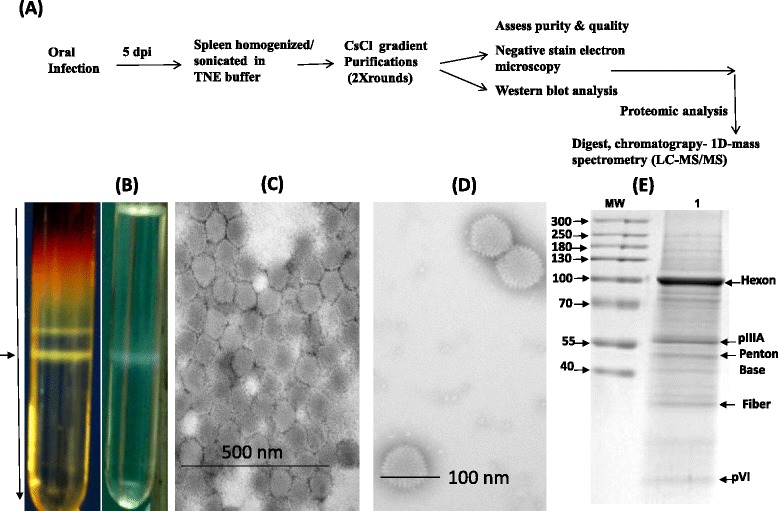
**Purification of TAdV-3 virions.** **A** *Strategy for enrichment and purification of TAdV-3 virions*. Flow diagram depicts the strategy. **B** CsCl2 purification of TAdV-3. The lower band containing mature viruses (left panel) subjected to second round of CsCl (right panel). **C**–**E** *Analysis of TAdV-3 virions purity.* Electron micrograph of CsCl purified TAdV-3 negatively stained with 2% aqueous phosphotungstic acid (Direct magnification 100000X) (**C**) and (direct magnification 150000X) (**D**). **E** *Analysis of TAdV-3 polypeptides*. Purified TAdV-3 proteins were separated by 10–15% SDS-PAGE and analyzed by Western blot using turkey anti-TAdV-3 serum [4,9] and peroxidase-linked goat anti-turkey IgG antibodies (right panel). The identified polypeptides (lane 1) are depicted. Molecular weight markers (MW) in kDa are shown on the left of the panel.

Due to difficulty in propagating turkey adenovirus 3 in cell culture system, TAdV-3 was propagated in six to 8 week old turkeys. TAdV-3 virions were purified from spleens of turkeys inoculated orally with an avirulent vaccine strain of TAdV-3 [4,9] (Figure 1A). Following CsCl_2_ density gradient purification, two distinct bands were observed, the upper band (present at lower density) containing capsid and the lower band (at higher density, between 1.25 and 1.35) containing complete infectious viruses (Figure 1B, left panel). The lower band was subjected to second round of CsCl_2_ density gradient purification resulting in single band containing purified virions (Figure 1B, right panel). Virion-enriched preparations were checked for quality by negative stain transmission electron microscopy (TEM) (Figures 1C and D). As seen, virions demonstrated uniform, intact TAdV-3 virus particles of 100 nm diameter. These TEM results were consistent with the quality and apparent purity reported earlier [33,35]. The purity of the virion preparation was also determined by Western blot analysis using turkey anti-TAdV-3 sera. As seen in Figure 1E, polypeptides of 96 K (hexon), 57 K (IIIa), 52 K (penton base), 29 K (fiber) and 24 K (pVI) were detected in CsCl_2_ purified TAdV-3 virions. These findings suggest that our enrichment procedure yielded a highly purified preparation of TAdV-3 virions.

### Protein composition of CsCl_2_ purified TAdV-3 virions

The protein composition of TAdV-3 virions was analyzed by the method of in-solution trypsin “digestion a gel-free approach” to MS that subject the entire sample to sequential one-dimensional reversed-phase chromatography coupled on-line to MS/MS analysis (1D-nanospray-LC-MS/MS). This method eliminates the problems reported with proteins that either enter gel poorly or extracted inefficiently from the gel slices. Our LC-MS/MS analysis revealed a total of 15 virus-encoded proteins packaged in the purified TAdV-3 virions. This included 13 proteins, which have been detected in human adenovirus 5 (HAdV-5) virions [16] (Table 1), a novel uncharacterized hypothetical viral protein designated as TaV3gp04 (Table 1, Additional file 1) and a non-structural viral protein (22 K) to be associated with TAdV-3 virions. In addition to TAdV-3 encoded viral proteins, interestingly 26 cellular proteins appeared to be associated with purified TAdV-3 virions (Table 2).

**Table 1:** TAdV-3 proteins identified by LC-MS/MS

	**LC-MS/MS**			
**Protein name**	**MW (kDa)** ^a^	**No. of peptides**	**Mascot score** ^**b**^	**Sequence coverage (%)** ^**c**^
pVI	24.98	146	4378	69
pIIIA	57.52	59	2377	96
pVII	13.20	163	1984	91
IVa2	42.36	39	1046	48
Penton Base	34.17	46	857	54
Hexon	101.65	42	659	23
pVIII	21.75	35	655	81
Fiber	29.13	18	620	29
**Hypothetical Protein (TaV3gp04)**	**13.32**	**17**	**452**	**35**
DBP	44.21	13	389	12
Sialidase	64.9	9	162	6
pX	6.15	9	137	29
Adenain	25.33	9	113	18
pTP	70.72	7	102	9
22 K	10.51	5	42	29

A novel virion-associated viral protein is shown in bold black.

^a^Theoritical molecular mass.

^b^A Mascot score ≥35 is significant (*p* < 0.05).

^c^Sequence coverage is based on peptides with an unique sequence.

**Table 2:** Cellular proteins associated with purified TAdV-3 virions identified by LC-MS/MS

	**LC-MS/MS**			
**Protein name**	**MW (kDa)** ^a^	**No. of peptides**	**Mascot score** ^b^	**Sequence coverage (%)** ^c^
Actin	42.36	56	607	38
TAR DNA binding protein 43	44.90	22	656	24
Tublin beta5	50.28	25	535	21
Tubulin alpha-1A	50.78	19	444	26
LUC7 like1	47.60	28	482	28
Tublin beta3	50.09	25	338	20
High mobility group protein B1	23.08	13	349	34
High mobility group protein B2	23.98	24	227	24
78 kDa glucose-regulated protein	72.08	21	135	5
Myeloid protein 1	36.41	14	240	23
Desmin	53.30	16	212	11
Cathelicidin-3	16.61	5	146	32
Protein PML	35.99	13	158	18
Vimentin	53.16	16	157	17
Splicing factor U2AF	28.19	7	154	20
Collagen alpha-1 (VI) chain	110.0	5	146	32
Elongation factor 1-alpha	49.48	6	143	15
Protein syndesmos	33.44	4	140	3
Ferritin	17.13	8	129	22
Serine/arginine-rich splicing factor 5a	27.15	2	76	7
Fibronectin	276.017	3	76	2
Gallinacin-2	7.49	2	75	12
Cathespsin B	38.47	3	70	9
Hemoglobinsubunit beta	16.62	6	64	19
L-amino acid oxidase	59.08	3	56	3
Hemoglobin subunit alpha-A	15.10	3	42	17

^a^Theoritical molecular mass.

^b^A Mascot score ≥35 is significant (*p* < 0.05).

^c^Sequence coverage is based on peptides with an unique sequence.

### Protein composition of proteinase K treated CsCl purified TAdV-3 virions

**Figure 2:**
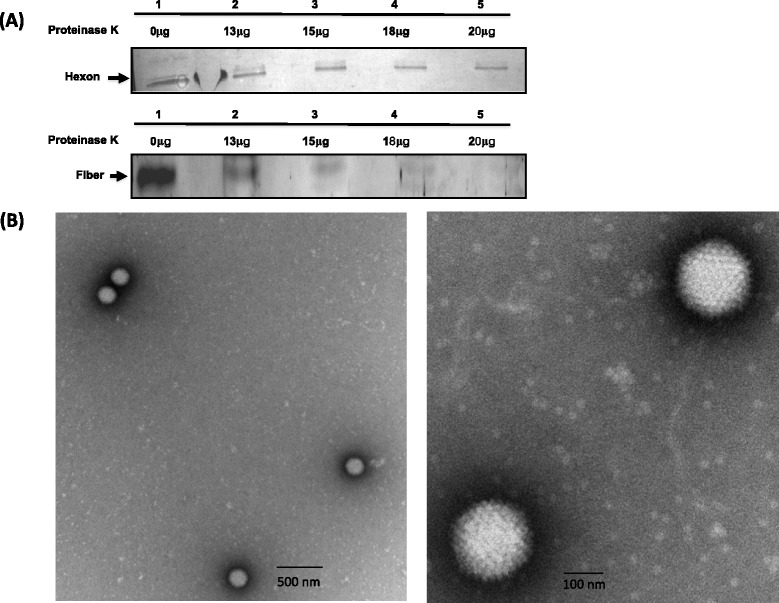
**Proteinase K digestion of purified TAdV-3 virions.** **A** Proteins from purified TAdV-3 untreated (lane 1) or treated (lanes 2–5) with indicated amounts of proteinase K were separated by 10–15% SDS-PAGE, transferred to nitrocellulose and probed by Western blot using anti-TAdV-3 serum. The hexon protein and the fiber protein are depicted by an arrow. Concentration of proteinase K in μg is indicated on top of the panels. **B** Purified TAdV-3 treated with 20 μg of proteinase K were negatively stained with 2% aqueous phosphotungstic acid and analyzed by transmission electron microscopy. (Direct magnification 50000×, left panel) and (direct magnification 150000×, right panel).

To determine if the host proteins are actually incorporated into the virions, the purified TAdV-3 virions were treated with proteinase K (20 μg/mL) and subjected to another round (third round) of CsCl_2_ purification. The proteinase K treated and untreated, purified virions were then analysed by Western blotting. Proteinase K treatment degrades fiber protein protruding from the capsid but does not degrade hexon protein not protruding from the capsid. As seen in Figure 2A, hexon protein could be detected in proteinase K treated or untreated TAdV-3 virions. In contrast, fiber protein could only be detected in untreated virions, but not in proteinase K treated virions. Moreover, TEM analysis suggested that the virions were intact and maintained virion integrity after proteinase K treatment and CsCl_2_ density gradient purification (Figure 2B).

**Figure 3:**
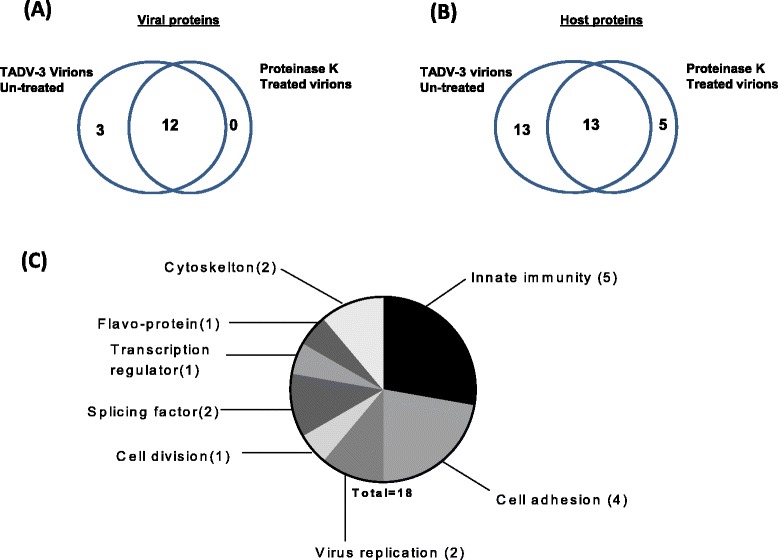
**Identification of viral and host virion proteins.** Venn diagrams of viral (**A**) and host proteins (**B**) detected in TAdV-3 untreated and proteinase K-treated samples by in-solution tryptic digestion followed by analysis using 1D-liquid chromatography combined with a mass spectrometer (LC-MS/MS). **C** Eighteen host-incorporated proteins identified in purified proteinase treated TAdV-3 virions in the presence of proteolytic digestion are classified based on their known functions.

The LC-MS/MS analysis of proteinase K treated CsCl_2_ density gradient purified TAdV-3 virions identified eleven virus-encoded proteins (hexon, pVI, pVII, penton base, pVIII, sialidase, IIIA, adenain, pX, IVa2 and DBP) previously reported to be in other adenoviruses (Table 3 and Figure 3A) [16]. In addition, a novel viral protein TaV3gp04 remains an integrated part of proteinase K treated TAdV-3 virions (Table 3, Additional file 1). As expected, peptides representing fiber protein were not detected in proteinase K treated TAdV-3 virions. In addition, pTP and 22 K virion proteins were not detected in proteinase K treated TAdV-3 (Table 3). The high mascot scores and number of peptides observed for hexon, pVI and pVII presumably reflect the fact that they are perhaps the most abundant proteins in the TAdV-3 particles.

**Table 3:** TAdV-3 proteins identified by LC-MS/MS after proteinase K treatment

	**LC-MS/MS**				
**Protein name**	**MW (kDa)** ^a^	**pK** ^**b**^	**No. of peptides**	**Mascot score** ^**c**^	**Sequence coverage (%)** ^**d**^
Hexon	101.653	+	71	1327	37
PVI	24.989	+	15	560	32
PVII	13.201	+	24	467	70
**Hypothetical Protein(TaV3gp04)**	**13.32**	**+**	**10**	**165**	**36**
Fiber	-	-	-	-	-
Penton Base	34.179	+	13	153	19
PVIII	21.75	+	8	135	12
Sialidase	64.9	+	2	84	10
IIIA	57.52	+	10	83	10
Adenain	25.33	+	2	55	6
PX	6.15	+	1	39	13
IVa2	42.36	+	2	35	2
DBP	44.21	+	1	24	2
pTP	-	-	-	-	-
22 K	-	-	-	-	-

A novel virion-associated viral protein is shown in bold black.

^a^Theoritical molecular mass.

^b^pK, proteinase K treatment +.

^c^A Mascot score ≥35 is significant (*p* < 0.05).

^d^Sequence coverage is based on peptides with an unique sequence.

Interestingly only 18 host proteins were exclusively detected in proteinase K treated TAdV-3 virions (Table 4 and Figure 3B). Notably, thirteen of these host proteins were the same as detected in the untreated TAdV-3 virions (Table 4, Figure 3B) indicating that these proteins are part of the TAdV-3 virions. Among these proteins, promyelocytic leukemia protein (PML) isoform X6 (Additional file 2), collagen alpha-1(VI) chain (Additional file 3), haemoglobin subunit alpha (Additional file 4) and haemoglobin subunit beta (Additional file 5) appeared abundant. The PML protein appears as abundant as viral structural protein pVIII or penton base peptide. In addition, five host proteins namely, vitronectin, collagen alpha-3 (VI) chain, collagen alpha-2 (VI) chain, tyrosine protein phosphatase and turkey heterophil peptide 2 (THP-2) were only detected in proteinase K treated TAdV-3 virions.

**Table 4:** Cellular proteins associated with purified TAdV-3 virions after proteinase K treatment

	**LC-MS/MS**					
**Protein name**	**Mass (kDa)** ^a^	**No. of peptides**	**Mascot score** ^b^	**Sequence coverage (%)** ^**c**^	**Protein function**	**Reported in other viruses**
**Protein PML isoform X6**	**48.4**	**8**	**141**	**10**	**Innate immunity**	-
**Collagen alpha-1 (VI) chain**	**110**	**17**	**101**	**7**	**Cell adhesion**	-
Vitronectin	52.2	4	100	5	Cell adhesion	SIV^21^, KSHV^29^
**Hemoglobin subunit alpha –A like**	**15.5**	**5**	**99**	**24**	**Innate immunity**	**Corona** ^**26**^ **, Influenza** ^**20**^
Collagen alpha-3 (VI) chain	340	9	92	10	Cell adhesion	-
Collagen alpha-2 (VI) chain	110	5	91	5	Cell adhesion	Influenza^20^
**Ferritin**	**17.1**	**4**	**90**	**20**	**Virus replication**	**HCV** ^**24**^ **, sHEV** ^**25**^
**Elongation factor 1-alpha**	**47.6**	**2**	**69**	**3**	**Virus replication**	**HIV** ^**13**^
**Hemoglobin subunit-beta like**	**16.3**	**8**	**66**	**27**	**Innate immunity**	**CSFV** ^**45**^
Tyrosine protein phosphatase	68	2	63	2	Cell division	-
Antimicrobial peptide THP-2	7.6	7	64	48	Innate immunity	-
**Splicing factor U2AF**	**28.7**	**5**	**60**	**9**	**Splicing factor**	**KSHV** ^**29**^
**Serine/arginine splicing factor 5α**	**30.1**	**1**	**43**	**3**	**Splicing factor**	**Influenza** ^**20**^
**TAR DNA binding protein 43**	**45.0**	**3**	**40**	**2**	**Transcription**	**HSV** ^**14**^ **,RSV** ^**23**^
**L-amino acid oxidase**	**59.08**	**4**	**40**	**7**	**Flavoprotein**	**-**
**Gallinacin-2**	**7.6**	**4**	**39**	**26**	**Innate immunity**	**-**
**Tubulin alpha-1A**	**50.9**	**4**	**32**	**4**	**Cytoskeleton**	**HIV** ^**13**^ **, Influenza** ^**11**^ **, ASFV** ^**28**^
**Actin**	**42.2**	**3**	**32**	**5**	**Cytoskeleton**	**HIV** ^**13**^ **, Influenza** ^**11**^ **, ASFV** ^**28**^

Protein subsets identified by LC-MS/MS with/without protease treatment are shown in bold black.

^a^Theoritical molecular mass.

^b^A Mascot score ≥30 is significant (*p* < 0.05).

^c^Sequence coverage is based on peptides with an unique sequence.

Functional classification of the identified proteins revealed that many of these proteins participate in a common molecular pathway (Table 4 and Figure 3C) and are involved in innate immunity, cell adhesion, cytoskeleton organization and virus replication.

### Validation of cellular proteins incorporated into TAdV-3 virions

**Figure 4:**
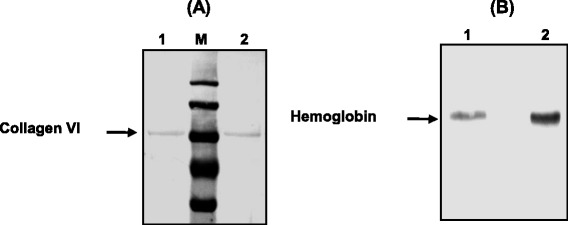
**Western blot analysis of host proteins in TAdV-3.** Proteins from the proteinase K untreated purified TAdV-3 virions (panels **A**, **B**, lane 1) and proteinase K (20 μg incubated in 1 mL of MNT buffer) treated purified TAdV-3 virions (panels **A**, **B**, lane 2) were separated by 10–15% SDS-PAGE, transferred to nitrocellulose and analyzed by Western blot using anti-collagen alpha-1(VI) chain serum (panel **A**) and anti-haemoglobin serum (panel **B**). Molecular weight markers (Lane M).

Non availability of turkey host protein specific antisera made it difficult to verify the packaging of host proteins in TAdV-3 virions. However, human collagen alpha-1(VI) peptides showed 70% identity to turkey collagen alpha-1(VI) and chicken collagen alpha-1(VI) (Additional file 6). In addition, human haemoglobin peptides demonstrated 75% identity to turkey haemoglobin alpha and chicken haemoglobin alpha, 50% identity to turkey haemoglobin beta and 66% identity to chicken haemoglobin beta proteins (Additional file 7). Therefore, we attempted to determine the incorporation of collagen alpha-1(VI) and haemoglobin in purified TAdV-3 using Western blot assays. As shown in Figure 4, anti-collagen alpha-1 (VI) serum detected collagen alpha-1 (VI) chain specific band in proteinase K untreated TAdV-3 (panel A, lane 1). Similar protein could be detected in proteinase K treated purified TAdV-3 (panel A, lane 2). Anti-haemoglobin serum detected haemoglobin specific band in proteinase K untreated TAdV-3 (panel B, lane 1). Similar protein band could be detected in proteinase K treated purified TAdV-3 (panel B, lane 2).

## Discussion

Viruses exploit multiple host proteins for successful entry, establishment of infection, replication, and immune evasion. For a better understanding of the TAdV-3-host interactions, we performed a comprehensive analysis of the protein content of TAdV-3 virions, using a LC-MS/MS based proteomic approach. To the best of our knowledge, incorporation of host proteins in adenovirus has not been reported so far.

The proteomic analysis of CsCl_2_ purified TAdV-3 identified a total of 13 virion proteins and 18 host proteins. Earlier, proteomic analysis has not reported the detection of host proteins in purified HAdV-5 virions [15,16]. It is possible that the observed host proteins identified by proteomic analysis of CsCl_2_ purified TAdV-3 virions may not be actually incorporated in the purified virions but are loosely associated on the outside of the TAdV-3 virion capsids. Since proteinase K treatment has been traditionally used to remove any contaminating protein from the surface of enveloped viruses [33,35], we used protease treatment of non-enveloped TAdV-3 to remove the potential contaminating proteins. Several lines of evidence validate the approach and suggest that proteinase K treatment of TAdV-3 appears successful in removing contamination proteins. 1) Intact virions could be detected by TEM after proteinase K treatment of TAdV-3. 2) Western blot analysis of protease K treated TAdV-3 detected hexon protein but not fiber protein (protruding from the capsid). 3) The fiber and 22 K (non structural protein) could not be detected by MS analysis of proteinase K treated TAdV-3. 4) Only 18 of the 26 host proteins could be identified in proteinase K treated TAdV-3.

Interestingly, all major viral proteins were identified in proteinase K treated virions (Table 3) except viral pTP, possibly due to its low abundance and least mascot score values observed (Table 1). Overall sequence coverage observed for different viral peptides ranged from 2 to 70%, with the majority between 10 and 35%.

Earlier, sequence analysis of turkey adenovirus-3 identified a hypothetical protein ORF 4 (named TaVgp04) [7], which appears to be conserved in raptor adenovirus-1 [36] and South polar skua adenovirus [37]. In contrast, a hypothetical hydrophobic protein was identified in frog adenovirus 1 [8], which shows no similarity to similar proteins identified in turkey adenovirus 3 [7] and raptor adenovirus 1 [36]. Our results suggest that an ORF4 of TAdV-3 encodes a structural protein TaVgp04, which is incorporated into virion capsid (Additional file 1). In addition, this is the first report to suggest the existence of TaVgp04 as a structural protein in siadenoviruses particularly of avian origin.

The proteomic analysis of proteinase K treated purified virions identified eleven cellular proteins incorporated in TAdV-3, which have been identified in other viruses (Table 4). In addition, proteomic analysis identified seven host proteins incorporated in TAdV-3 virions (Table 4), which have not been identified so far in any other virus. Interestingly, of the 18 detected host proteins, five of the proteins were only detected in proteinase K treated TAdV-3. It is possible that high abundance non-specific proteins might have masked the detection of these proteins in virions not treated with proteinase K that are truly virion associated, but present in low copy numbers.

Though earlier reports have demonstrated the packaging of viral [38] or non viral RNAs [39] into purified adenovirus, recent reports have not described the detection of any cellular protein in purified Lizard adenovirus-2 [40], a member of *Atadenovirus* genus and purified HAdV-5, a prototype of *Mastadenoviru*s genus [15]. The absence of a cellular protein packaged in purified adenovirus virions could be due to variety of reasons. As stated, the difference could be due to the technique used for analysis [15]. Alternatively, it is possible that packaging of the cellular proteins may be dependent on the type of adenovirus (TAdV-3, a prototype of *Siadenovirus* genus) and origin of cells used for virus cultivation [15].

The host proteins packaged inTAdV-3 are known to play important roles in enhancing the cell-to-cell spread of virus, transcription and virus replication (Table 4, Figure 3). For example, extracellular matrix (collagen) has been shown to increase infectious Sindbis virus titers from BHK cells by enhancing post-infection cell survival [41]. In another study, rotavirus-induced PI3K activation resulted in prolonged adherence of infected cells to collagen and increased virus production [42]. Similarly, extracellular matrix vitronectin has been reported to enhance the growth of human adenovirus19 (HAdV-19) [43].

However, the incorporation of antiviral host defense factors including, protein PML, haemoglobin and antimicrobial peptide (THP-2) into TAdV-3 virions is particularly intriguing. All of these host defence factors have been implicated in establishing antiviral environments. Recent studies have implicated PML in maintaining host antiviral defence and revealed different strategies developed by viruses to disrupt PML nuclear bodies [44-46]. In addition, protein PML has been shown to be important for the inhibition of adenovirus replication [47]. Similarly, avian antimicrobial peptide THP-2, a member of beta-defensin family is effector of the innate defence system and play key functions during host defence by generating vigorous cytokine response [48,49]. On the other hand, a novel role of haemoglobin in innate immunity has been recently reported for classical swine fever virus (CSFV) [50] as silencing of haemoglobin expression using siRNA promoted CSFV growth and replication, whereas overexpression of haemoglobin antagonized CSFV replication and growth by triggering IFN signalling [50].

Although TAdV-3 grows efficiently in spleen of infected turkey, virus grows poorly in primary or established cell lines. It is tempting to speculate that integration of certain established antiviral host restriction factors into viral particles may play a role in determining TAdV-3 replication “in vitro”. Additional studies need to be performed in order to investigate whether these proteins are functionally required for virus entry, replication and pathogenesis. Future availability of reagents and a reliable cell culture system to grow TAdV-3 should make it possible to determine the role of individual host restriction factor in TAdV-3 replication.

## Additional files

Additional file 1:
**Novel viral protein TaV3gp04.** Sequence showing peptides detected in LC-MS/MS. Additional file 2:
**Amino acid sequence of protein PML.** Sequence showing peptides detected in LC-MS/MS. Additional file 3:
**Amino acid sequence of collagen alpha-1(VI) chain.** Sequence showing peptides detected in LC-MS/MS. Additional file 4:
**Amino acid sequence of haemoglobin subunit alpha-A-like.** Sequence showing peptides detected in LC-MS/MS. Additional file 5:
**Amino acid sequence of haemoglobin subunit beta-like.** Sequence showing peptides detected in LC-MS/MS. Additional file 6:
**Amino acid sequence similarity of collagen alpha-1(VI) peptides.** i) Human (accession number NP_001839) ii) turkey (accession number XP_003207392.1) iii) chicken (accession number CAA45788.1) peptide residues. Numbers to the left are the peptide position in coding sequence of the protein. Matched peptides shown in color. Additional file 7:
**Amino acid sequence similarity of Haemoglobin peptides.** i) Human haemoglobin (accession number NP_000549.1) ii) Chicken haemoglobin alpha (accession number NP_001004376.1) iii) Chicken haemoglobin beta (NP_990820.1) iv) Turkey haemoglobin alpha (accession number XP_003210796.1) V) Turkey haemoglobin beta (accession number XP_003203315.1) peptide residues. Numbers to the left are the peptide position in coding sequence. Matched peptides shown in colour.
