# Supplementary material for: Proteomic analysis of purified turkey adenovirus 3 virions
Source: Vet Res. 2015 Jul 9;46(1):79. doi: 10.1186/s13567-015-0214-z (PMC4497381; doi:10.1186/s13567-015-0214-z)
Supplement: Additional file 1: — Novel viral protein TaV3gp04. Sequence showing peptides detected in LC-MS/MS. [file 13567_2015_214_MOESM1_ESM.docx]

**Additional file 1 Novel viral protein TaV3gp04**

**Source CDS^1^ Protein Sequence^2^**

Turkey 115 TaV3gp04

MPFFYLVGAGSAYCERCKEVCEKRRKRSTTRTTRTK**RSKPSHLQYVR**YYPGTVVPVGWDGTDKPVTVTR**IPDYWTYDR**AVSSR**QSNTVVPVNTSGENPTVVAVPR**LLRKRKASDM

^1^ Featuring length of coding sequence of the protein. ^2^ Matched peptides shown in bold black.
