# Supplementary material for: Proteomic analysis of purified turkey adenovirus 3 virions
Source: Vet Res. 2015 Jul 9;46(1):79. doi: 10.1186/s13567-015-0214-z (PMC4497381; doi:10.1186/s13567-015-0214-z)
Supplement: Additional file 2: — Amino acid sequence of protein PML. Sequence showing peptides detected in LC-MS/MS. [file 13567_2015_214_MOESM2_ESM.docx]

**Additional file 2 Amino acid sequence of protein PML**

**Source CDS ^1^ Protein Sequence ^2^**

PNSSRPAFSYHPRLPPSPSSSFSPRRTAETKAKRPAQRPEMPGSPEAPRTSGPQEDGPAAKEPGTAPGPSRCPTPSGLLGEGDFQFLLCEGCKQESLNLKLLTCLPLCLGCLRENKPIGQCPVCQTPIPQPDGIPDVDNVLXYRR**ISSGGLSCCR**CRREAAAMWCSECEEFLCPGCFEDHQWFFKKRSHEARKVEELRAESAHRFLEGTKK**SCSLFCSSPRHTEQGHVTSIFCR**KCEKPLCCSCALLDAQHSSFYCDIRTEIQRQQDELAELGQELAQQRGGFEASRAALQEK**AAQLEAAGHGVR**ELVRQRVEQLVRLIRREEEELLATVGRKQEKGHKELEKELWRVEAVLRRMEAGERLVEKMGLYATEQEVMDMQPFIKDALEELRRQRPAADGELEVHEDFAECRARLQALTECI

Turkey 419 PREDICTED: protein PML

isoform X6 [Gallus gallus]

^1^ Featuring length of coding sequence of the protein. ^2^ Matched peptides shown in bold black.
