# Supplementary material for: Proteomic analysis of purified turkey adenovirus 3 virions
Source: Vet Res. 2015 Jul 9;46(1):79. doi: 10.1186/s13567-015-0214-z (PMC4497381; doi:10.1186/s13567-015-0214-z)
Supplement: Additional file 3: — Amino acid sequence of collagen alpha-1(VI) chain. Sequence showing peptides detected in LC-MS/MS. [file 13567_2015_214_MOESM3_ESM.docx]

**Additional file 3 Amino acid sequence of collagen alpha-1(VI) chain**

**Source CDS^1^ Protein Sequence^2^**

MGLHDSFLALLLLLGGAWAQQAEINARVLRAQDCPVDLFFVLDTSESVALRVKPFGDLVAQVKDFTNRFIDKLTERYFRCDRFLAWNAGALHYSDSVVIIKDLTAMPSGRAELK**NSVSAINYIGKGTHTDCAIKQGIER**LLLGGSHLKENKYLIVVTDGHPLEGYKEPCGGLDDAANEAK**HLGIKVFSVAISPHHLDQR**LNIIATDHAYRRNFTATSLKPTRDLDVEETINNIIEMIKDNMEQSCCSFECHPPRGPPGPPGDPGHEGERGKPGLPGQKGDAGDPGRPGDMGPVGYQGMKGDKGSRGEKGSRGAKGAKGEKGKRGIDGIDGMKGEAGYPGLPGCKGSPGFDGTQGPPGPKGDPGAYGPKGGKGEPGEDGKPGRQGIPGSPGEKGAPGNRGEPGPLGETGDEGSPGADGPPGERGSNGERGPPGSPGDRGPRGDLGEPGPPGDQGREGPLGPPGDQGEPGPPGPKGYRGDDGPRGNEGPKGSPGAPGLPGDPGLMGERGEDGPPGNGTIGFPGAPGQQGDRGDPGINGTKGYVGPKGDEGEAGDPGNDNPTAGPSGIKGAKGHRGPEGRPGPPGPVGPPGPDECEILDIIMKMCSCCECTCGPVDLLFVLDSSESIGLQNFQIAKDFIIKVIDRLSKDER**VKFEPGESR**VGVVQYSHNNTQELVAMGDANIDNIGALKQAVKNLKWIAGGTHTGEALQFSKENLLRRFTSNNNVAIVITDGRSDTLRDRTPLTSLCEVTPVVSLGIGDIFRNNPNPDQLNDIACLGMPR**RQGLSIQR**DNYAELLDDSFLQNITSYVCREKKCPDYTCPITFANPADIMLLVDSSTSVGSKNFDTTKNFVKRLAER**FLEASKPAEDSVR**VSVVQYSGRNQQKVEVPFQRNYTVIAKAVDNMEFMNEATDVNAALQYIMGLYQRSSRSGAKKKVLVFSDGNSQGITARAIERTVQEVQQAGIEVYVLAVGSQVNEPNVRVLVTGKSTNYDVAYGERHLFRVPDYTSLLRGVFYQTVSRKIAVD

Turkey 1019 PREDICTED: collagen alpha-1(VI)

chain-like[Meleagris gallopavo]

^1^ Featuring length of coding sequence of the protein. ^2^ Matched peptides shown in bold black.
