# Supplementary material for: Proteomic analysis of purified turkey adenovirus 3 virions
Source: Vet Res. 2015 Jul 9;46(1):79. doi: 10.1186/s13567-015-0214-z (PMC4497381; doi:10.1186/s13567-015-0214-z)
Supplement: Additional file 5: — Amino acid sequence of haemoglobin subunit beta-like. Sequence showing peptides detected in LC-MS/MS. [file 13567_2015_214_MOESM5_ESM.docx]

**Additional file 5 Amino acid sequence of haemoglobin subunit beta-like**

**Source CDS^1^ Protein Sequence^2^**

M**VHWSAEEK**QLITGLWGK**VNVADCGAEALARLLIVYPWTQR**FFASFGNLSSPTAILGNPMVRAHGKKVLTSFGDAVKNLDNIKKSFAQLSKLHCDK**LHVDPENFR**LLGDILIVVLASHIGKDFTPASQAAWQKMVRVVAHALAHEYH

Turkey 147 PREDICTED: haemoglobin

subunit beta-like

[Meleagris gallopavo]

^1^ Featuring length of coding sequence of the protein. ^2^ Matched peptides shown in bold black.
