# Supplementary figures and images for: Proteomic analysis of purified turkey adenovirus 3 virions
Source: Vet Res. 2015 Jul 9;46(1):79. doi: 10.1186/s13567-015-0214-z (PMC4497381; doi:10.1186/s13567-015-0214-z)

Additional file 6 Amino acid sequence similarity of collagen alpha-1(VI) peptides


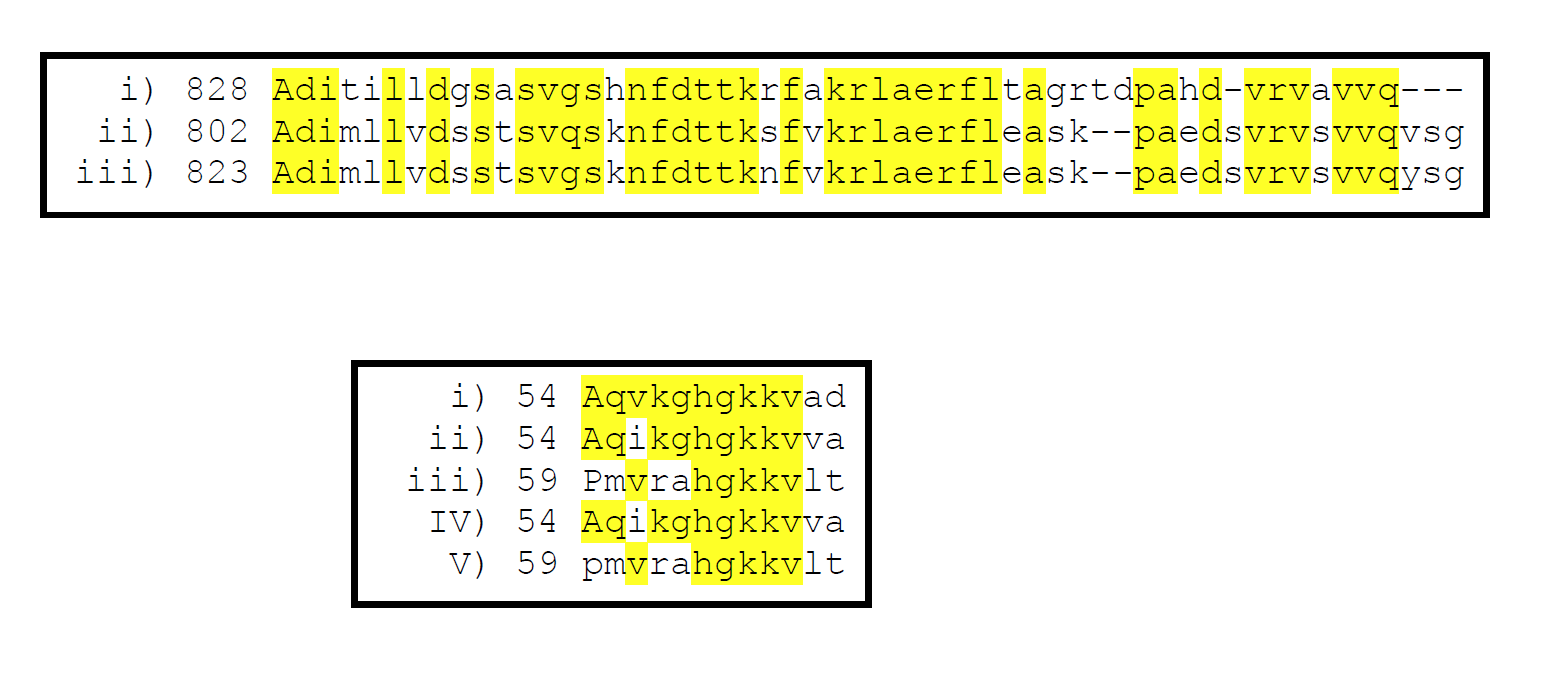

Supplement: Additional file 6: — Amino acid sequence similarity of collagen alpha-1(VI) peptides. i) Human (accession number NP_001839) ii) turkey (accession number XP_003207392.1) iii) chicken (accession number CAA45788.1) peptide residues. Numbers to the left are the peptide position in coding sequence of the protein. Matched peptides shown in color. [file 13567_2015_214_MOESM6_ESM.docx]

Additional file 7 Amino acid sequence similarity of Haemoglobin peptides


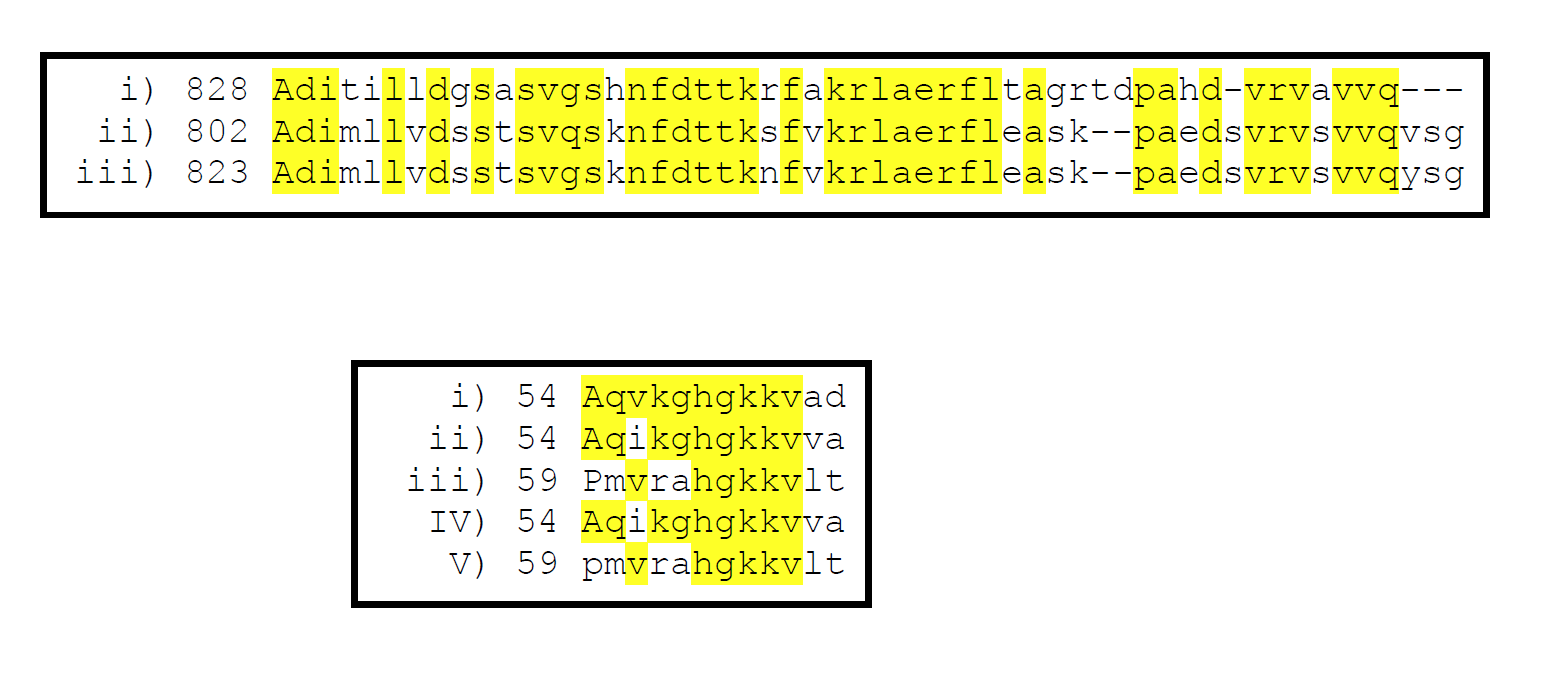

Supplement: Additional file 7: — Amino acid sequence similarity of Haemoglobin peptides. i) Human haemoglobin (accession number NP_000549.1) ii) Chicken haemoglobin alpha (accession number NP_001004376.1) iii) Chicken haemoglobin beta (NP_990820.1) iv) Turkey haemoglobin alpha (accession number XP_003210796.1) V) Turkey haemoglobin beta (accession number XP_003203315.1) peptide residues. Numbers to the left are the peptide position in coding sequence. Matched peptides shown in colour. [file 13567_2015_214_MOESM7_ESM.docx]
